# Multitargeted Effects of *Plantago ovata* Ethanol Extract in Experimental Rat Streptozotocin-Induced Diabetes Mellitus and Letrozole-Induced Polycystic Ovary Syndrome

**DOI:** 10.3390/ijms26104712

**Published:** 2025-05-14

**Authors:** Lia-Oxana Usatiuc, Raluca Maria Pop, Surd Adrian, Marcel Pârvu, Mădălina Țicolea, Ana Uifălean, Dan Vălean, Laura-Ioana Gavrilaș, Szabo Csilla-Enikő, Loredana Florina Leopold, Floricuța Ranga, Florinela Adriana Cătoi, Alina Elena Pârvu

**Affiliations:** 1 Pathophysiology, Department 2—Functional Sciences, Faculty of Medicine,“Iuliu Hațieganu” University of Medicine and Pharmacy, 400012 Cluj-Napoca, Romania; lia.usatiuc@umfcluj.ro (L.-O.U.); madalina.ticolea@umfcluj.ro (M.Ț.); ana.uifalea@umfcluj.ro (A.U.); adriana.catoi@umfcluj.ro (F.A.C.); parvualinaelena@umfcluj.ro (A.E.P.); 2 Pharmacology, Toxicology and Clinical Pharmacology, Department 2—Functional Sciences, Faculty of Medicine, “Iuliu Hațieganu” University of Medicine and Pharmacy, 400012 Cluj-Napoca, Romania; 3 Pediatric Surgery and Orthopedics, “Iuliu Hațieganu” University of Medicine and Pharmacy, 400012 Cluj-Napoca, Romania; 4 Department of Taxonomy, Faculty of Biology and Geology, “Babes-Bolyai” University, 400012 Cluj-Napoca, Romania; marcel.parvu@ubbcluj.ro; 5 Surgery Department, “Iuliu Hațieganu” University of Medicine and Pharmacy, 400012 Cluj-Napoca, Romania; valean.d92@gmail.com; 6 Faculty of Nursing and Health Sciences, Department 2, “Iuliu Hațieganu” University of Medicine and Pharmacy, 23 Marinescu Street, 400337 Cluj-Napoca, Romania; laura.gavrilas@umfcluj.ro; 7 First Pediatric Clinic, Department of Mother and Child, “Iuliu Hațieganu” University of Medicine and Pharmacy, 400012 Cluj-Napoca, Romania; csilla.szabo@umfcluj.ro; 8 Faculty of Food Science and Technology, University of Agricultural Sciences and Veterinary Medicine, 3–5 Calea Mănăștur, 400372 Cluj-Napoca, Romania; loredana.leopold@usamvcluj.ro (L.F.L.); florica.ranga@usamvcluj.ro (F.R.)

**Keywords:** *Plantago ovata*, diabetes mellitus, polycystic ovary syndrome, oxidative stress, inflammation

## Abstract

Polycystic ovary syndrome (PCOS), a common and multifactorial endocrine disorder in reproductive-aged women, is strongly associated with insulin resistance (IR) and type 2 diabetes mellitus (T2DM), and also affects up to one in four women with type 1 diabetes mellitus (T1DM). The current study explored the potential of *Plantago ovata *(*P. ovata*) seed ethanol extract (POEE) to modulate oxidative stress (OS), inflammatory responses, metabolic profiles, and hormonal levels in rat Streptozotocin (STZ)-induced DM and Letrozole (LET)-induced PCOS. Phytochemical analysis measured total phenolic content (TPC) and total flavonoid content (TFC) using HPLC-DAD-ESI MS for compound identification. POEE’s antioxidant activity was evaluated in vitro through DPPH, H_2_O_2_, FRAP, and NO scavenging assays. Rats received POEE, metformin, or Trolox (TX) for 10 days. PCOS confirmation was achieved via ultrasound and histopathology. Serum levels of OS markers (TOS, TAC, OSI, MDA, AOPP, 8-OHdG, NO, 3-NT, AGEs, and SH), inflammatory markers (NF-κB, IL-1β, IL-18, Gasdermin D, and IL-10), metabolic parameters (fasting blood glucose, lipid profile, and liver enzymes), and hormone levels (LH, FSH, estrogen, testosterone, and insulin) were assessed. Additionally, the Triglyceride–Glucose index (TyG) and HOMA-IR were calculated. POEE had a medium content of polyphenols and a good in vitro antioxidant effect. In vivo, POEE administration in diabetic rats led to a reduction in OS markers and an increase in antioxidant levels, alongside decreases in inflammatory cytokines, blood glucose levels, and transaminase activity and improvements in lipid profile. In the PCOS model, POEE treatment effectively reduced total OS and lowered levels of LH, FSH, and testosterone, while elevating estrogen concentrations and reducing insulin resistance. These therapeutic effects were dose-dependent, with higher doses producing more pronounced outcomes, comparable to those observed with metformin and TX treatment.

## 1. Introduction

Diabetes mellitus (DM) is a chronic metabolic disorder resulting from abnormalities in insulin secretion, insulin action, or both. It is fundamentally characterized by persistent hyperglycemia and is commonly associated with varying degrees of dysregulation in carbohydrate, lipid, and protein metabolism [1,2].

Polycystic ovary syndrome (PCOS) is the most prevalent endocrine disorder affecting women of reproductive age worldwide. It is characterized by hyperandrogenism, oligo-anovulation, and polycystic ovarian morphology. The condition is driven by a complex interplay of genetic, environmental, and lifestyle factors [3].

The relationship between type 2 diabetes mellitus (T2DM) and PCOS is defined by a strong bidirectional association, underpinned by shared pathophysiological mechanisms—most notably, insulin resistance (IR) [4]. Up to one in four women with type 1 diabetes mellitus (T1DM) also meet the diagnostic criteria for PCOS. A recognized pathophysiological mechanism in this context is systemic hyperinsulinemia, resulting from exogenous insulin administration, which bypasses hepatic first-pass metabolism [5].

Oxidative stress (OS)—an imbalance favoring reactive oxygen species (ROS) over antioxidant defenses—has emerged as a significant contributor to the pathogenesis of numerous chronic diseases, including both DM and PCOS. In T2DM, hyperglycemia strongly induces OS through multiple pathways, accelerating the development of diabetic complications [6]. Increasing evidence also implicates OS in PCOS, linking it to hyperandrogenism, IR, ovulatory dysfunction, and chronic inflammation [7]. Given its established role in both conditions, OS likely serves as a convergent mechanism contributing to the increased risk of DM observed in women with PCOS. Specifically, OS-induced IR and inflammation may synergistically drive the progression and comorbidity of both disorders.

Chronic low-grade inflammation is being increasingly recognized as a key feature in the pathogenesis of T2DM, significantly contributing to both the development of IR and the progression of complications. Among the critical regulators of this inflammatory response, the NLR family pyrin domain containing 3 (NLRP3) inflammasome has emerged as a central mediator in DM. Furthermore, recent evidence suggests that inflammation in PCOS is not merely secondary to metabolic disturbances, but plays an active role in the syndrome’s pathophysiology and associated comorbidities. Inflammatory processes worsen IR and hyperandrogenism, impair ovulatory function, and contribute to the increased risk of metabolic complications in women with PCOS [8].

*Plantago ovata* (*P. ovata*), commonly known as blond psyllium, belongs to the *Plantaginaceae* family and is native to the Mediterranean region, North Africa, and West Asia. It is best known for its high soluble fiber content, particularly arabinoxylan polysaccharides, which are the principal active constituents of its seed husks. These fibers exert multiple physiological effects, including increasing intestinal viscosity, delaying gastric emptying, and reducing glucose absorption in the small intestine—actions that enhance insulin sensitivity and modulate the gut microbiota. Beyond its fiber content, *P. ovata* also contains various bioactive phytochemicals, such as flavonoids (e.g., luteolin-7-O-glucoside and apigenin-7-O-glucoside), iridoid glycosides (e.g., aucubin and catalpol), and phenolic acids (notably acteoside), all of which contribute to its antioxidant, anti-inflammatory, hypoglycemic, and antimicrobial properties. These features support its broader therapeutic potential beyond its laxative effect, including roles in cholesterol reduction, weight management, and the relief of irritable bowel syndrome symptoms [9,10].

This study aimed to evaluate the effects of *P. ovata* ethanol extract (POEE) on OS, inflammation, and disruptions in glucose and lipid metabolism in a Streptozotocin (STZ)-induced rat model of DM. In parallel, the effects of POEE on OS, inflammation, IR, androgen levels, and the hypothalamic–pituitary–gonadal axis were assessed in a Letrozole (LET)-induced rat model of PCOS. Additionally, the extract underwent phytochemical screening, in vitro antioxidant activity assessment, and an evaluation of potential hepatotoxicity.

## 2. Results

### 2.1. Plant Extract Phytochemical Characterization

**Figure 1:**
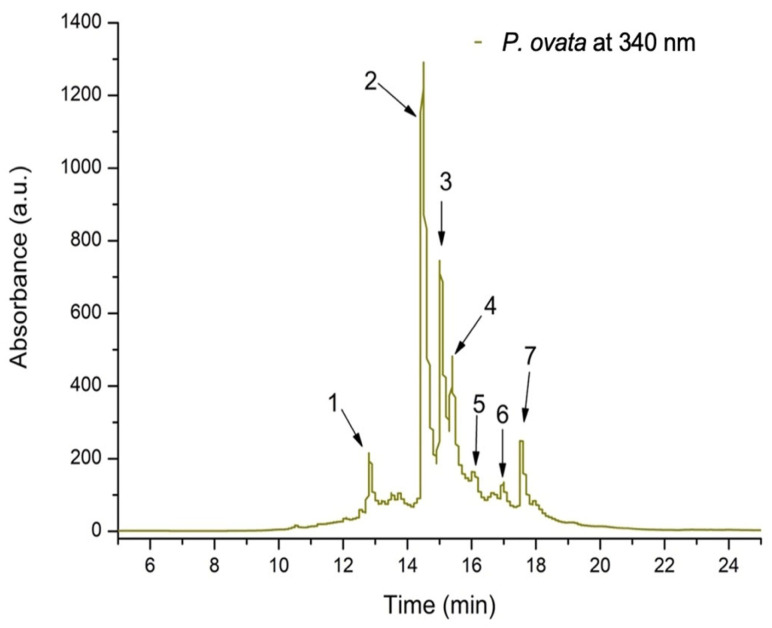
The HPLC chromatogram of phenolic content from *P. ovata* ethanolic extract at 340 nm.

The total phenolic content (TPC) of POEE, determined using the Folin–Ciocâlteu method, was 6.2 mg GAE/100 g d.w. plant material, and its total flavonoid content (TFC), determined using the aluminium chloride method, was 4.79 mg QE/g d.w. plant material. To determine the polyphenolic compounds in POEE, an optimized HPLC/MS method was used. In total, seven compounds were identified, out of which seven were flavonoles, and one compound belonged to the flavones subclass (luteolin-diglucoside). Among flavone compounds, quercetin derivatives and kaempferol derivatives were the most represented ones (Figure 1, Table 1).

POEE contains the following two kaempferol derivatives: kaempferol-rutinoside, corresponding to peak 1, with *m*/*z* 595, and kaempferol-diglucoside, corresponding to peak 7, with *m*/*z* 611. It also contains four quercetin derivatives, including quercetin-xylosyl-rutinoside corresponding to peak 2, with *m*/*z* 743,303, quercetin-rutinoside (Rutin) corresponding to peak 3, with *m*/*z* 611, quercetin-glucoside corresponding to peak 4, with *m*/*z* 465, and quercetin-glucuronide corresponding to peak 6, with *m*/*z* 479, and one luteolin derivative (luteolin-diglucoside) corresponding to peak 5, with *m*/*z* 611.

**Figure 2:**
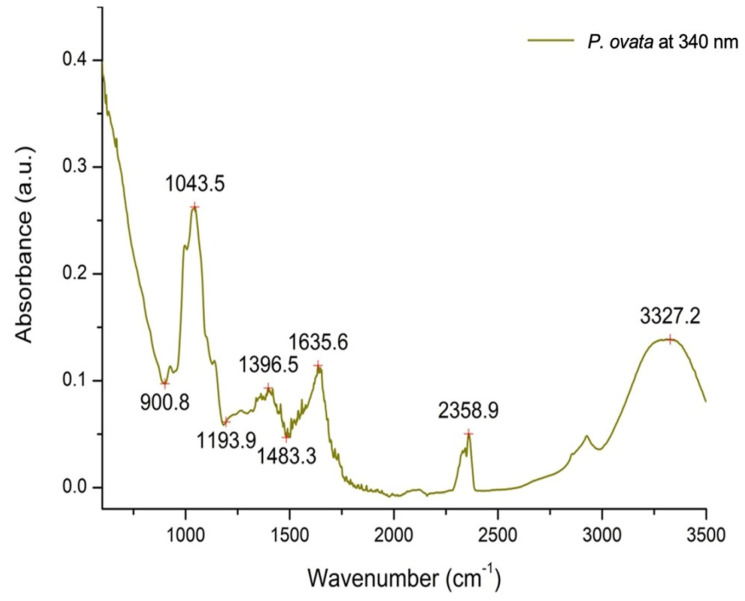
The FTIR analysis of phenolic content from *P. ovata* ethanolic extract at 340 nm.

The FTIR fingerprint of POEE presents the absorption bands characteristic of compounds extracted with the 70% ethanol extraction solvent (Figure 2). The FTIR spectrum revealed several characteristic absorption bands corresponding to major functional groups like 3327.2 cm^−1^ (O-H stretching vibrations), 2358.9 cm^−1^ (C≡C or C≡N stretching), 1635.6 cm^−1^ (C=C stretching vibrations), 1396.5 cm^−1^ (C–H bending or symmetric stretching of carboxylate groups), and 1043 cm^−1^ (C–O stretching vibrations) [11,12].

### 2.2. Assessment of the In Vitro OS Markers

POEE exhibited good in vitro antioxidant activity in all the tests. DPPH assay showed moderate antioxidant activity, with the result being between 50 and 100 μg TE/mL (*p* < 0.001). POEE H_2_O_2_ scavenging activity (*p* < 0.001) and FRAP (*p* < 0.001) results were better as compared to Trolox (TX). POEE NO scavenging activity was also better as compared to quercetin (*p* = <0.001) (Table 2).

### 2.3. Evaluation of In Vivo Antioxidant Effects of P. ovata Ethanol Extract

When comparing the OS parameters in the STZ model to the CONTROL model, there were significant increases in the oxidants, including total oxidative status (TOS), oxidative stress index (OSI), malonyldialdehide (MDA), and AOPPs (advanced oxidation protein products) (*p* < 0.001), 8-hydroxydeoxyguanosine (8-OHdG), NOx (nitric oxide), 3NT (3-nitrotyrosine), and advanced glycation end products (AGEs) (*p* < 0.05), respectively. The antioxidant defense markers, total antioxidant capacity (TAC) and thiols (SH), were significantly decreased (*p* < 0.05) (Table 3).

By comparing the POEE effects with the STZ group, we found that TOS and OSI were significantly reduced by POEE100% and POEE50% (*p* < 0.001), with POEE25% having a smaller inhibitory effect on TOS and OSI (*p* < 0.05). The efficiency of POEE100% and POEE50% was comparable to that of metformin and TX. MDA was significantly reduced by all three POEE concentrations, metformin, and TX (*p* < 0.001). AOPPs were lowered by POEE100% and POEE50% (*p* < 0.001), POEE25% exhibited a small inhibitory effect (*p* < 0.05), and metformin and TX had no effect. 8-OHdG was lowered by POEE100% and POEE50% (*p* < 0.01), and POEE25% had little inhibitory activity (*p* < 0.05). Metformin and TX had no statistically significant effect on 8-OHdG (*p* > 0.05). AGEs were reduced only by the POEE100% (*p* < 0.05) and metformin (*p* < 0.01) treatments. NO was decreased significantly by POEE100% and POEE50% (*p* < 0.01), with POEE25% having a small effect (*p* < 0.05). 3NT was decreased by all three concentrations in the same manner (*p* < 0.001), and the effect was better than that of metformin (*p* < 0.05). TX had no important effect on 3NT (*p* > 0.05). The extract improved antioxidant defense by increasing TAC very significantly for all three concentrations (*p* < 0.001), and POEE100% and POEE 50% also caused an increase in SH (*p* < 0.001) (Table 3).

In the POEE groups, Pearson’s correlation analysis revealed a strong positive correlation between MDA, TOS, and OSI levels (*p* < 0.001) and between AOPPs, TOS, OSI, and NO (*p* < 0.001), a moderate positive correlation between 8-OHdG, AGEs, and 3NT (*p* < 0.05), and a strong negative correlation between TAC, MDA, TOS, and OSI (*p* < 0.001) and between SH and MDA, TOS, and OSI (*p* < 0.001), respectively.

When comparing the OS parameters in the LET to the CONTROL, there were significant increases in the oxidants TOS, OSI, MDA, AOPPs, and NOx (*p* < 0.001), 8-OHdG (*p* < 0.01), and 3NT (*p* < 0.05), respectively. The antioxidant defense markers TAC and SH were significantly decreased (*p* < 0.001) (Table 4).

By comparing the effects of POEE100% with the LET group, we found that TOS and OSI were reduced (*p* < 0.05), but not as much as by TX treatment (*p* < 0.001). MDA and AOPPs were significantly reduced by POEE100% (*p* < 0.01). TX had a stronger inhibitory effect on MDA (*p* < 0.001), and metformin had better inhibitory activity on AOPPs (*p* < 0.001). 8-OHdG was also lowered by POEE 100% and TX (*p* < 0.001), with metformin having a smaller inhibitory effect (*p* < 0.01). AGEs, NOx, 3NT, and SH were not influenced by POEE100% (*p* > 0.05). The extract improved antioxidant defense by increasing TAC (*p* < 0.001) (Table 4).

In all of the PCOS groups, Pearson’s correlation analysis revealed a strong positive correlation between MDA, TOS, OSI, AOPP, 8-OHdG, AGE, and 3NT levels (*p* < 0.001) and a strong negative correlation between TAC and MDA, TOS, OSI, MDA, AOPPs, AGEs, and 8-OHdG (*p* < 0.001) and between SH and TOS (*p* < 0.01), OSI (*p* < 0.01), MDA (*p* < 0.05), and AGEs (*p* < 0.05), respectively.

### 2.4. In Vivo Inflammatory Markers Analysis

The inflammatory response was assessed by quantifying nuclear factor kappa B-p65 (NF-κB-p65), Interleukin (IL-1β), Interleukin-18 (IL-18), and Gasdermin D as markers of NLRP3 inflammasome activation. Compared to the control group, the STZ group exhibited statistically significant increases in IL-1β and IL-18 (*p* < 0.001), as well as NF-κB p65 and Gasdermin D (*p* < 0.01).

Treatment with POEE, metformin, and TX significantly reduced NF-κB p65 levels (*p* < 0.05). All three POEE concentrations, metformin, and TX significantly decreased IL-1β and IL-18 levels (*p* < 0.001) (Table 5). Gasdermin D levels were significantly reduced by POEE 100% and 50% (*p* < 0.01).

When comparing the inflammatory response obtained in the LET group to CONTROL, we found statistically significant increases in NFkB-p65, IL-1B, and IL-18 (*p* < 0.01) and in the anti-inflammatory cytokine IL-10 (*p* < 0.001). POEE100% lowered NFkB-p65, IL-1B, and IL-18 (*p* < 0.01), and IL-10 was also significantly reduced (*p* < 0.001). IL-1B (*p* < 0.01), IL-18 (*p* < 0.01). and IL-10 (*p* < 0.001) were decreased in the metformin group. In comparison to LET group, the TX group demonstrated significant decreases in NF-κB p65 and IL-1β (*p* < 0.01), and moderate but significant decreases in IL-18 and IL-10 (*p* < 0.05) (Table 6).

### 2.5. Assessment of In Vivo Metabolic and Hepatoprotective Activities

The successful induction of DM in the STZ group was confirmed by a significant elevation in GLU levels compared to the CONTROL group (*p* < 0.001). Animals failing to reach the hyperglycemia threshold of 250 mg/dL were excluded from the study. POEE100% resulted in a significant decrease in GLU (*p* < 0.01), comparable to the effect of metformin (*p* < 0.01). POEE50% and POEE25% elicited a less pronounced reduction in GLU, which was not statistically significant (*p* > 0.05) (Table 7).

Evaluations of lipid profiles revealed significant increases in TG and TC in the STZ group relative to the CONTROL group. POEE100% and POEE50% significantly lowered TG levels (*p* < 0.05), demonstrating effects similar to TX administration (*p* < 0.05). Neither POEE25% nor metformin significantly altered TG levels (*p* > 0.05). The Triglyceride–Glucose index (TyG) was elevated in STZ-treated animals (*p* < 0.01). POEE100% and POEE50% produced a marginal but significant decrease in the TyG index (*p* < 0.05), whereas POEE25%, metformin, and TX had no significant impact (*p* > 0.05). All POEE concentrations and metformin significantly reduced TC (*p* < 0.001), while TX had no substantial effect (*p* > 0.05) (Table 7).

Liver injury in the STZ group was characterized by significant increases in AST and ALT levels (*p* < 0.001) and a decrease in the AST/ALT ratio (*p* < 0.05) compared to the CONTROL group. POEE100% and POEE50% significantly lowered AST and ALT levels (*p* < 0.01), whereas POEE25% showed no effect on liver enzymes (*p* > 0.05). Metformin reduced only ALT levels (*p* < 0.05), and TX had no significant effect on liver injury markers. POEE, metformin, and TX did not significantly alter the AST/ALT ratio compared to the STZ group (*p* > 0.05) (Table 7).

STZ-induced DM was associated with a significant increase in BW (*p* < 0.001). Treatment with POEE100%, metformin, and TX significantly reduced BW compared to the STZ group (*p* < 0.01), while the POEE50% and POEE25% concentrations produced a smaller, yet significant, decrease in BW (*p* < 0.05) (Table 7).

### 2.6. Effect of P. ovata Ethanolic Extract on Sex Hormones, Serum Insulin, and HOMA-IR in Rat LET-Induced PCOS

When comparing the insulin levels in the LET group to those in the CONTROL, we found statistically significant increases in insulin (*p* < 0.001), GLU (*p* < 0.05), and the HOMA-IR index (*p* < 0.001). POEE100%, metformin, and TX significantly reduced insulin levels (*p* < 0.001). GLU was significantly lowered by POEE100% (*p* < 0.001), while metformin and TX had less inhibitory activity *p* < 0.05). HOMA-IR was significantly reduced by POEE100%, metformin, and TX (*p* < 0.001) (Table 8).

Treatment with LET caused a significant increase in gonadotropins levels, follicle-stimulating hormone (FSH), and luteinizing hormone (LH) (*p* < 0.01), respectively, when compared to the CONTROL, while estrogen levels were lowered (*p* < 0.001), confirming the presence of positive feedback on the pituitary hormones. Treatment with POEE100% and TX significantly lowered LH (*p* < 0.05), with metformin having no important effect (*p* > 0.05). FSH was also decreased by POEE, but the difference was not statistically significant. Metformin and TX had no effect on FSH levels. Estrogen was increased by all treatments, POEE100%, metformin, and TX (*p* < 0.001), respectively. In the LET group, testosterone levels were increased (*p* < 0.05) when compared to the CONTROL. POEE100% lowered testosterone levels (*p* < 0.05), while metformin and TX had no effect when compared to LET (Table 8).

### 2.7. Principal Component Analysis of Serum Biomarkers

**Figure 3:**
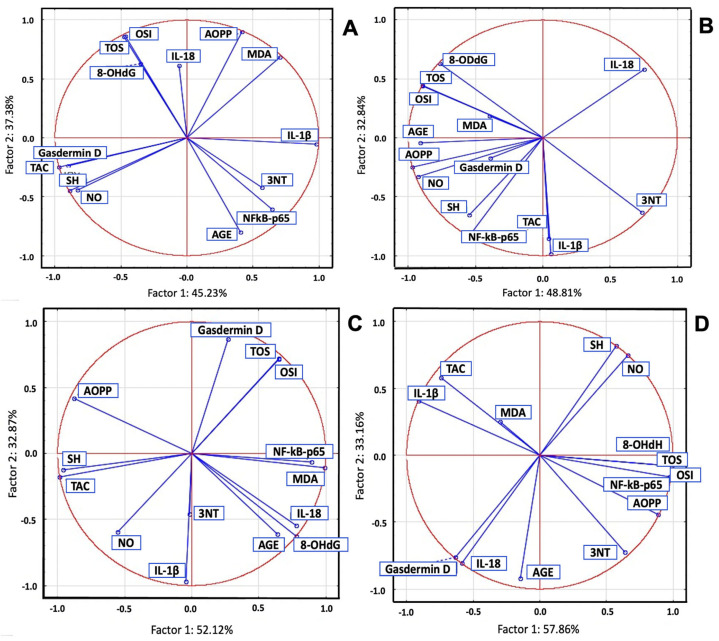
OS and inflammatory markers PCA results: (**A**) STZ group; (**B**) POEE100%; group; (**C**) POEE50% group; and (**D**) POEE25% group.

In the STZ group, significant positive correlations were observed between inflammatory and oxidant markers, as well as significant negative correlations with TAC and SH. PCA revealed a positive correlation between IL-18 and OSI, TOS, 8-OHdG, AOPPs, and MDA. NF-κB p65 and IL-1β were strongly correlated with 3NT and AGEs, while Gasdermin D was correlated with TAC, SH, and NO (Figure 3).

In the POEE100% group, IL-1β, NF-κB p65, and Gasdermin D were correlated with antioxidant system parameters, TOS, OSI, MDA, AOPP, 8-OHdG, NO, and AGEs, and with 3NT. In the POEE50% group, NF-κB p65, IL-1β, and Gasdermin D were correlated with TOS, OSI, AGEs, 8-OHdG, and MDA, while IL-1β was strongly correlated with 3NT and moderately correlated with antioxidant system parameters and NO. In the POEE25% group, NF-κB p65 was strongly correlated with TOS, OSI, MDA, AOPPs, and 8-OHdG; Gasdermin D and IL-18 were correlated with AGEs; and IL-1β was correlated with TAC and MDA (Figure 3).

**Figure 4:**
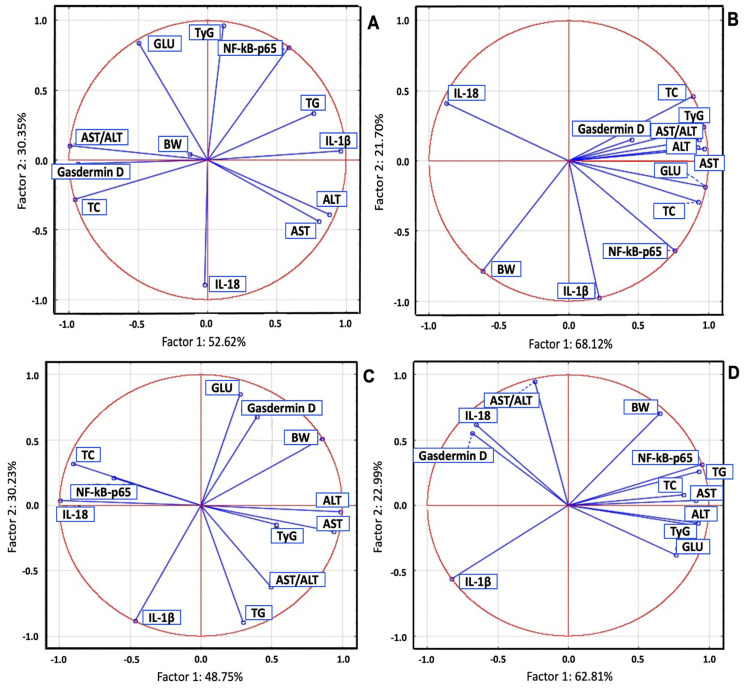
OS, metabolic, and liver injury marker PCA results in STZ-induced DM in rats: (**A**) STZ group; (**B**) POEE100% group; (**C**)POEE50% group; and (**D**) POEE25%.

In the STZ group, PCA revealed strong correlations between IL-1β and AST, ALT, and TG, while NF-κB p65 was strongly correlated with TyG and GLU, and Gasdermin D was correlated with the AST/ALT ratio, TC, and BW. In the POEE100% group, Gasdermin D and NF-κB p65 were strongly correlated with TG, GLU, TyG, AST, ALT, and the AST/ALT ratio, while IL-18 and IL-1β were correlated with BW. In the POEE50% group, strong correlations were observed between IL-1β and liver injury markers; NF-κB p65 and GLU and TG; and liver injury enzymes and TG. Gasdermin D was correlated with liver injury enzymes, GLU, BW, and TyG, while NF-κB p65 and IL-18 were correlated with TC, and IL-1β was correlated with TG and the AST/ALT ratio. In the POEE25% group, NF-κB p65 was correlated with GLU, TC, TG, TyG, liver injury enzymes, and BW, while Gasdermin D and IL-18 were correlated with the AST/ALT ratio (Figure 4).

**Figure 5:**
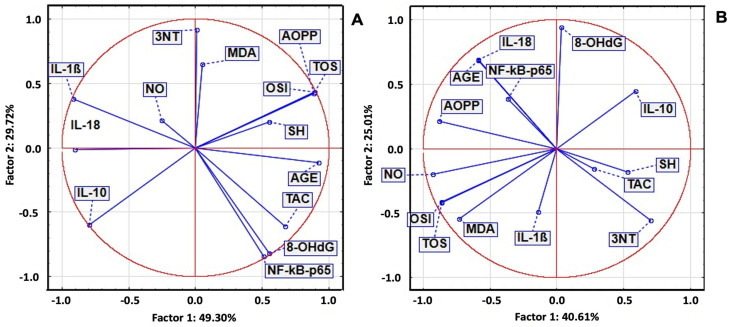
OS and inflammatory markers PCA result in LET-induced PCOS in rats: (**A**) LET group and (**B**) POEE100% group.

In the LET group, between inflammatory and oxidant markers, we noticed a statistically significant positive correlation and a significant negative correlation with antioxidant system parameters. PCA analysis indicated that a positive correlation between NFkB-p65, IL-18, and IL-1B with OSI, TOS, 8-OHdG, AOPPs, MDA, AGEs, and NO was present. In the same group, IL-10 was correlated with TAC, SH, and 3NT. In the POEE 100% group, NFkB-p65 was correlated with antioxidant system parameters, TOS, OSI, AOPPs, AGEs, and 8-OHdG, while IL-1B and IL-18 were correlated with NO and 3NT (Figure 5).

**Figure 6:**
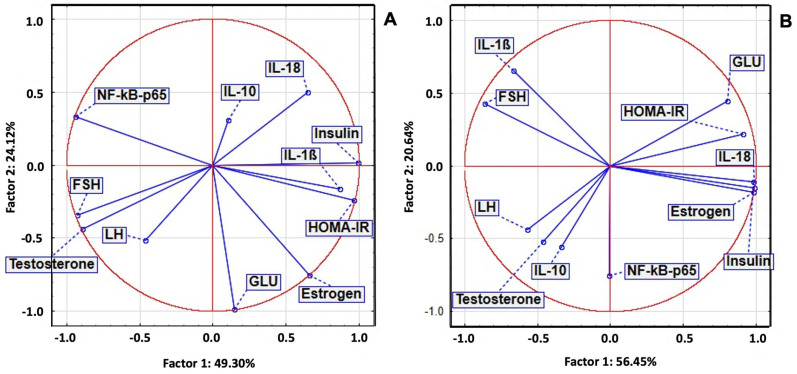
Hormonal and inflammatory markers PCA results in LET-induced PCOS in rats: (**A**) LET group and (**B**) POEE100% group.

In the LET group, between inflammatory and hormonal markers, we noticed a statistically significant positive correlation between NFkB-p65 and IL-18 with insulin, GLU, HOMA-IR, and estrogen. PCA analysis indicated that a positive correlation between IL-1B, FSH, and LH, while IL-10 was moderately correlated with insulin, GLU, HOMA-IR, estrogen, LH, and FSH. In the POEE 100% group, NFkB-p65 was correlated with FSH, LH, and testosterone, while IL-1B and IL-18 were correlated with insulin, GLU, HOMA-IR, and estrogen (Figure 6).

### 2.8. Histological and Follicular Changes in Letrozole-Induced Hyperandrogenized Rat Ovaries Following Treatment with P. ovata Ethanolic Extract

**Figure 7:**
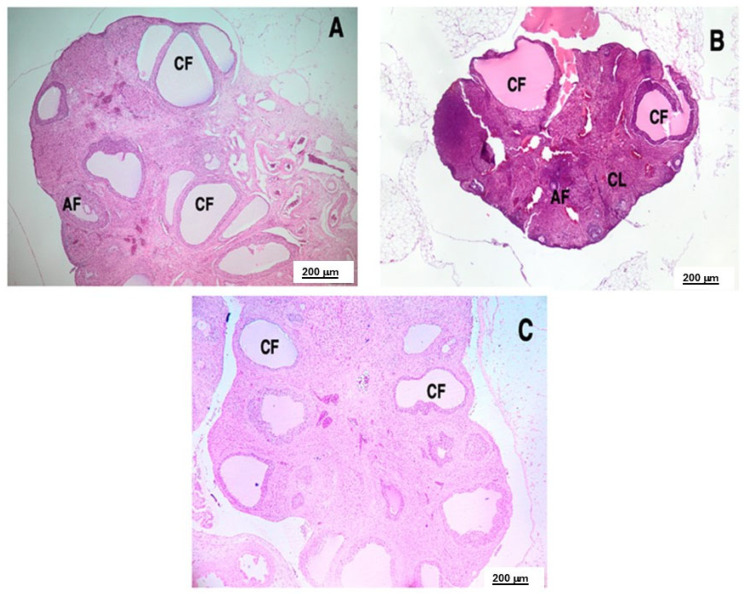
Effect of POEE treatment on histology of LET-induced ovary. (**A**) LET group, (**B**) LET + POEE100% group, and (**C**) LET + M group. AF, antral follicle; CL, corpus luteum; CF, cystic follicle.

Microscopic examination of H&E-stained ovarian tissue from LET-exposed rats demonstrated marked alterations in ovarian architecture. The LET group exhibited a prevalence of large cystic formations, coupled with a notable deficiency or lack of corpora lutea (Figure 7A). Within these cysts, granulosa cells were diminished and organized into only two or three layers along the basement membrane, resulting in the formation of atretic follicles characterized by a prominent central lumen. However, the administration of POEE and metformin led to a discernible amelioration of ovarian histology, shown by a decrease in or elimination of cystic structures and a reduction in atretic antral follicles. Furthermore, the previously dense and clustered granulosa cell layer within the larger follicles exhibited signs of restoration, and the development of corpora lutea was observed following treatment with POEE and metformin (Figure 7B,C).

### 2.9. Ultrasonography Findings

**Figure 8:**
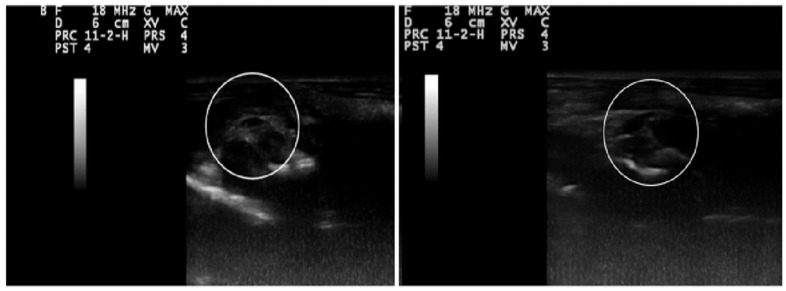
Ultrasound images of ovaries of PCOS animals: bilateral enlarged ovaries with oblonged multiple cystic follicles.

On the tenth day of the study, ultrasound imaging validated the development of ovarian cysts in the LET-treated group. The scans revealed a notable increase in ovarian size, accompanied by the presence of numerous elongated cystic formations, thereby confirming the induction of cystic ovaries (Figure 8).

## 3. Discussion

Diabetes mellitus (DM) is a frequent non-communicable chronic disease. For the two types of DM, T1D and T2D, respectively, the currently available therapies include insulin and various oral antidiabetic drugs, but most of them have negative side effects. For this reason, herbal medicine has proposed that medicinal plants that have been used in the treatment of DM should be scientifically evaluated in order to obtain new complementary antidiabetic drugs [13]. OS, chronic inflammation, and accompanying metabolic disorders are mechanisms involved in the pathogenesis of DM and its associated complications [14]. This study revealed that POEE had a beneficial impact on metabolic imbalances, OS, and chronic inflammation—core pathological features of DM and its complications—in diabetic rats induced with STZ.

The *Plantago* genus, comprising over 200 species, has long been recognized in traditional medicine for its broad spectrum of therapeutic properties. Among these, *P. ovata*—commonly known as *Psyllium*—is particularly valued for its high soluble fiber content and bioactive compounds. Recent studies have highlighted the significant antioxidant activity of *P. ovata*, attributed to its rich content of polyphenols and flavonoids, which help to neutralize ROS and reduce oxidative damage in tissues [15].

Polyphenols are regarded as the most abundant and widespread plant secondary metabolites. The TPC and TFC results for POEE are consistent with the findings of other *Plantago* species [16], but it has to be considered that the use of different extraction solvents is a factor influencing TPC and TFC values [17,18]. The presence of other polar compounds extracted in the investigated ethanolic extract was also evident after the FTIR analysis. Accordingly, the strong absorption band at 3327.2 cm^−1^ can be attributed to -OH stretching in the phenolic compounds, water, or can indicate the presence of possibly extracted polysaccharides. Next, the absorption band at 1635 cm^−1^ confirms the presence of phenolics, flavonoids, or proteins in the extract and is characteristic of C=C stretching in the aromatic ring or Amide I [11]. Because of the high mucilage content characteristic of *P. ovata*, which is mainly composed of polysaccharides, the peak at 1396.5 cm^−1^ can be assigned to C-H bending in the sugar rings [12]. The peak at 1043.5 cm^−1^ could be due to C-O stretching in the sugar moieties in glycosides or alcohol groups [19].

Referring to phenolic compounds, the main peaks identified in POEE were flavonoids, mainly from the flavonol subclass, including derivatives of quercetin and kaempferol, as well as flavones, represented by luteolin-diglucoside. Previous studies have also identified the presence of quercetin and kaempferol derivatives, including rutin, in *P. ovata* [9,10]. Quercitin exhibits strong antioxidant activity by scavenging free radicals, chelating metal ions, and enhancing the activity of antioxidant enzymes such as superoxide dismutase (SOD) and catalase [20]. Quercetin also shows significant anti-inflammatory effects, primarily through the inhibition of pro-inflammatory mediators like the TNF-α, IL-6, and NF-κB signaling pathways [21]. In addition, quercetin has demonstrated anti-diabetic potential, as it improves insulin sensitivity, modulates glucose metabolism, and protects pancreatic β-cells against OS [22,23,24]. Therapeutic effects of quercitin in animal models of PCOS have been also reported [25]. Kaempferol is a plant-derived flavonoid with notable antioxidant and anti-inflammatory effects [26,27]. Additionally, kaempferol has been reported to improve insulin sensitivity and helps to regulate blood glucose, making it relevant in managing metabolic disorders, with its effects being previously reported in STZ-induced diabetes in rats [28,29,30]. Previous studies have also reported the potential beneficial effects of kaempferol in the management of PCOS [31].

In DM, chronic hyperglycemia leads to long-term microvascular complications, including retinopathy, nephropathy, and neuropathy, as well as macrovascular complications primarily driven by atherosclerosis. Substantial evidence now indicates that OS, closely associated with DM, plays a central role in the pathogenesis of these vascular complications [32] As a result, targeting OS has become a key therapeutic strategy in DM, prompting extensive research into the antioxidant potential of various natural plant-derived compounds.

Numerous studies have demonstrated that plant polyphenols can mimic the actions of pharmacological agents, exhibiting notable antioxidant, anti-inflammatory, and hypoglycemic effects, while also enhancing the efficacy of conventional antidiabetic drugs through synergistic interactions [18,33]. These beneficial effects are largely attributed to the ability of phenolic compounds to mitigate OS by scavenging ROS and inhibiting their production [34,35]. In this context, POEE has shown promising in vitro antioxidant activity, which appears to be closely linked to its rich chemical composition.

To assess the in vivo effects of POEE, a STZ-induced T1DM rat model was employed [36]. STZ is a broad-spectrum antibiotic produced by *Streptomyces achromogenes* and a glucose analog that accumulates in pancreatic β-cells and causes their selective destruction [37,38]. T1DM was confirmed by significant hyperglycemia observed two days post-STZ administration.

In normal cells, there is a balance between the beneficial effects of ROS as signaling molecules and their harmful effects on cellular macromolecules such as lipids, proteins, and nucleic acids. Cells possess an antioxidant defense system comprising both enzymatic and non-enzymatic components that function synergistically to neutralize ROS [39]. OS arises when this balance is disrupted, typically due to excessive ROS production or a weakened antioxidant system. OS can result in cellular dysfunction, injury, inflammation, and even organ failure [39]. In DM, the ROS-induced oxidation of biomolecules contributes to long-term complications, even after glycemic control is achieved—a phenomenon known as “metabolic memory” [33]. Given that OS is intricately linked with impaired glucose and lipid metabolism in DM, numerous studies have investigated the effects of plant-based antioxidants.

Our findings revealed that the STZ-induced T1D model exhibited significant OS. Notably, OS biomarkers, including TOS, OSI, MDA, AOPPs, 8-OHdG, AGEs, NOx, and 3NT, were elevated, while TAC and SH were reduced from day two post-induction, compared to the control group. Treatment with POEE significantly decreased general oxidative markers (TOS and OSI) and markers of oxidative damage. Metformin, a known antioxidant, exhibited a comparable efficacy [40].

MDA is a key indicator of lipid peroxidation [14], and its reduction following POEE treatment indicates diminished ROS activity. Similarly, POEE lowered AOPPs, a marker of protein oxidation, which is relevant since AOPPs can induce inflammatory cytokine production in DM [41].

Oxidative DNA damage, as indicated by increased 8-OHdG levels, is associated with genomic instability and may lead to inheritable epigenetic alterations [33]. Flavonoid-rich diets have been shown to reduce 8-OHdG levels [42], supporting the observed decrease in 8-OHdG following POEE treatment, likely due to its flavonoid content.

In insulin-independent glucose-transporting cells, hyperglycemia promotes the non-enzymatic glycation of protein residues, contributing to elevated HbA1c and the formation of AGEs. These AGEs, exacerbated by OS, modify protein structure and function and trigger NF-κB activation, which promotes inflammatory gene expression and perpetuates metabolic memory [33,43]. POEE’s inhibition of AGE formation represents a significant antioxidant mechanism. Moreover, as metformin also inhibits AGEs, the synergistic action of POEE and metformin offers an enhanced therapeutic potential in advanced T1D.

NO, an unstable radical, is converted into nitrites and nitrates (NOx) [33,44]. Under normal conditions, low levels of NO act as antioxidants by scavenging ROS [35]. However, the hyperglycemia-induced expression of inducible nitric oxide synthase (iNOS) leads to excessive NO. POEE significantly reduced NOx, suggesting the inhibition of iNOS and/or enhanced free radical scavenging, consistent with known polyphenol activity [45,46]. Additionally, the observed decrease in the nitrosative stress marker 3NT suggests that POEE may help to reduce peroxynitrite (ONOO−) formation, a reactive nitrogen species implicated in tissue damage and mitochondrial dysfunction [47,48].

Antioxidants provide protection by limiting the damaging effects of ROS. In addition to endogenous enzymatic and non-enzymatic antioxidant defense, plant polyphenols play an important antioxidant role [33]. TAC measures total antioxidant activity, while SH groups, particularly protein SH, represent major plasma antioxidants [14,49]. POEE treatment significantly increased both TAC and SH levels. When combined with metformin, this effect was further enhanced, suggesting potential for synergistic antioxidant therapy.

In DM, OS and hyperglycemia initiate chronic inflammation, forming a vicious cycle of mutual reinforcement [35]. Disrupting this cycle is a therapeutic priority. The NF-κB pathway, particularly the p65:p50 heterodimer, governs inflammatory gene expression. ROS can activate NF-κB directly or through DNA damage [39,50]. POEE, rich in plant polyphenols, suppressed NF-κB p65 activity in a dose-dependent manner, especially at higher doses.

The NLRP3 inflammasome is a key driver of chronic inflammation and metabolic disturbances in diabetes, contributing to IR, β-cell damage, and related complications. Its activation involves a priming phase—triggered by hyperglycemia and OS—that upregulates NLRP3 and pro-inflammatory cytokines via NF-κB. This is followed by an activation phase initiated by factors like mitochondrial ROS, ER stress, and AGEs, leading to inflammasome assembly, caspase-1 activation, and cytokine maturation. The effector protein gasdermin D (GSDMD) facilitates pyroptosis by forming membrane pores, promoting the release of IL-1β and IL-18, thereby amplifying tissue inflammation and damage [51]. Plant polyphenols identified in our extract like quercitin and kaempferol have been reported to inhibit the activation of the NLRP3 inflammasome, thereby contributing to its anti-inflammatory effects. Quercetin has been shown to block NLRP3 activation by reducing ROS generation, inhibiting NF-κB signaling, and stabilizing mitochondrial function [52]. Similarly, kaempferol has been demonstrated to inhibit NLRP3 inflammasome assembly by interfering with the NLRP3/CASP1/GSDMD axis, modulating the interplay between the PPARγ and STAT1 pathways, and suppressing mitochondrial dysfunction [53]. POEE downregulated NLRP3 and its downstream effectors, further confirming its anti-inflammatory properties. Similar to metformin, which also suppresses NF-κB and NLRP3 activation, POEE demonstrated a dose-dependent efficacy evidenced by significant reductions in IL-1β, IL-18, GSDMD, and caspase-1 levels compared to untreated diabetic controls [35,51,54].

Recent studies have demonstrated that *P. ovata* extracts may serve as effective adjuncts in the oral management of T2DM [55,56,57,58]. The proposed mechanisms underlying their antihyperglycemic activity include the inhibition of intestinal glucose absorption and the enhancement of gastrointestinal motility. Additionally, the anti-diabetic properties of polyphenolic compounds—particularly quercetin derivatives, luteolin, and rutin—have been well-documented, supporting the therapeutic potential of *P. ovata* through its rich phytochemical profile [59,60,61]. POEE lowered the blood glucose concentration in STZ animals, having a dose-dependent hypoglycemic effect similar to that of metformin.

DM-induced dyslipidemia, characterized by elevated TG and TC, heightens the risk of lipid peroxidation and cardiovascular complications [33,62]. The TyG index, a surrogate marker of IR [63], was elevated in STZ-induced T1D, indicating the association of IR. In T1D, increased MDA covalently binds lipids and proteins in cell membranes, reducing insulin receptors’ internalization and the number of insulin-binding sites leading to IR [33]. Therefore, POEE treatment lowering the effect of the TyG mechanism may be reduction in hyperglycemia, hypertriglyceridemia, and MDA. These activities were also dose-dependent, with the highest dose being the most efficient.

OS and inflammation in DM can impair liver function, contributing to non-alcoholic fatty liver disease (NAFLD) and cirrhosis [35]. In STZ rats, elevated liver enzymes (AST and ALT) were significantly reduced by POEE, highlighting its hepatoprotective effect.

PCOS and DM share overlapping pathophysiological features, including IR, elevated androgen levels, chronic low-grade inflammation, and OS. In T2DM, PCOS is largely driven by peripheral IR, which leads to compensatory hyperinsulinemia and dyslipidemia. This interplay creates a self-reinforcing metabolic and hormonal feedback loop that increases the risk of developing diabetes [64]. In contrast, in T1DM, persistent hyperinsulinemia due to exogenous insulin administration, combined with inflammatory responses and ovarian dysfunction, may contribute to the emergence of PCOS-like symptoms [65]. A better understanding of these shared mechanisms can aid in refining diagnostic criteria and developing more effective management strategies for women affected by both conditions.

At present, PCOS treatment options mainly offer short-term relief and include symptom-targeted pharmacotherapy, lifestyle interventions, ovulation induction, anti-androgen agents, and the management of metabolic disturbances. However, these strategies do not address the root causes of the disorder and are often linked to adverse effects such as mood disorders, abnormal uterine bleeding, increased cardiovascular, thromboembolic, or hepatic risks [66]. As a result, there is a growing interest in complementary and alternative therapies that target the underlying disease mechanisms. Among these, plant-derived polyphenols have emerged as promising candidates for PCOS management [67].

The LET-induced PCOS rat model replicates key features of the human condition, including disrupted androgen–estrogen conversion. According to the Rotterdam criteria, PCOS diagnosis requires at least two of the following: oligo-anovulation, polycystic ovaries on ultrasound, and hyperandrogenism [68,69]. In this study, 21 days of LET administration induced PCOS in rats, confirmed by elevated testosterone and ovarian ultrasound findings. The observed hormonal alterations included reduced estrogen levels and elevated FSH and LH, accompanied by hyperinsulinemia, hyperglycemia, and an increased HOMA-IR index, all indicative of IR-associated DM.

The administration of POEE helped to restore hormonal balance by decreasing the levels of LH and FSH, while simultaneously elevating estrogen and reducing testosterone concentrations. These changes suggest a positive modulatory effect of POEE on the hypothalamic–pituitary–ovarian (HPO) axis. Metformin demonstrated a modest reduction in androgen levels and a slight elevation in estrogen concentrations; however, it did not significantly influence the levels of gonadotropins. Furthermore, POEE contributed to enhanced insulin sensitivity, as evidenced by reductions in circulating insulin, glucose levels and the HOMA-IR index. These improvements were less pronounced than those observed with metformin treatment. Existing research suggests that *P. ovata* may improve insulin sensitivity through modulated carbohydrate absorption and gut microbiota influence [70,71]. Furthermore, the improvement in insulin sensitivity and decreased HOMA-IR values may reflect an enhancement in insulin receptor signaling. This effect may be potentially mediated by flavonoid components like quercitin and kaempferol, which have been demonstrated to modulate key pathways such as PI3K/Akt and AMPK [72,73,74,75,76]. While Real et al. found no significant estrogenic or anti-estrogenic activity in *P. ovata* seed methanol extracts, direct anti-androgenic effects remain inconclusive. However, given that hyperinsulinemia can exacerbate hyperandrogenism, particularly in PCOS, *P. ovata*’s potential to enhance insulin sensitivity could indirectly influence androgen levels [70]. Moreover, its fiber-rich composition may influence the enterohepatic circulation of hormones and enhance the production of sex hormone-binding globulin (SHBG), contributing to the reduction in free androgens [77].

Rats with PCOS exhibited notable histopathological alterations in their ovarian tissue, characterized by cystic structures, increased atretic follicles, and a reduced number of corpora lutea, reflecting impaired folliculogenesis and anovulation. The administration of POEE and metformin markedly improved these abnormalities by minimizing cyst formation, lowering the incidence of follicular atresia. POEE also enhanced corpus luteum development, indicating a potential recovery of normal follicular maturation and ovulatory activity.

OS contributes significantly to the development of PCOS by disrupting the balance between ROS and antioxidant defenses. This imbalance is linked to IR, inflammation, and excess androgen production, all of which play key roles in PCOS pathophysiology. By impairing insulin signaling and ovarian hormone synthesis, OS perpetuates both metabolic and reproductive dysfunctions, making it a valuable target for therapeutic intervention [78]. In this model, PCOS was associated with increased oxidative biomarkers (TOS, OSI, MDA, AOPPs, 8-OHdG, AGEs, NOx, and 3NT) and decreased antioxidants (TAC and SH). POEE treatment showed moderate antioxidant effects, reducing several OS markers and enhancing TAC, though less effectively than metformin or TX. In PCOS, insulin-driven LH-mediated androgen production and cytochrome P450 activity contribute to ROS generation and OS [68,79]. Thus, POEE’s insulin-lowering effect likely underpins its antioxidant action. OS-induced mitochondrial DNA (mtDNA) damage can further promote inflammation and accelerate PCOS progression [80,81].

Chronic low-grade inflammation is now understood to be a critical factor in PCOS pathogenesis. Women with this condition present with elevated pro-inflammatory cytokines, dysregulated adipokine profiles, increased monocyte activation and endothelial dysfunction markers, and elevated AGEs [7]. This inflammatory state, exacerbated by OS, leukocyte activation, and potential gut microbiota dysbiosis, contributes to IR by disrupting insulin receptor signaling and amplifying OS. [6,68]. Within the ovarian microenvironment, inflammatory mediators disrupt normal folliculogenesis and steroidogenesis, promoting hyperandrogenism and anovulation [82]. The activation of inflammatory pathways, notably NF-κB and the NLRP3 inflammasome, further links immune activation to the endocrine and metabolic abnormalities characteristic of PCOS [83]. Our study aligns with these findings, showing increased levels of inflammatory cytokine levels that confirmed NLRP3 inflammasome activation. POEE markedly reduced NF-κB levels, lowered IL-1β and IL-18, and increased the levels of the anti-inflammatory cytokine IL-10.

When compared with other plant extracts, such as *Curcuma longa* (turmeric), which is rich in curcumin, and *Camellia sinensis* (green tea), known for its catechins, POEE demonstrated comparable antioxidant, anti-inflammatory, antidiabetic, and antiandrogenic efficacy. Curcumin has been shown to inhibit NF-κB activation and reduce cytokine levels in diabetic models, exerting positive effects on lipid profiles and IR, appearing to be a valuable treatment regimen for patients with PCOS [84,85]. Green tea polyphenols, especially the main components of (-)-epigallocatechin-3-gallate (EGCG) and (-)-epicatechin-3-gallate (ECG), possess many health-promoting and disease-preventing benefits, especially anti-inflammatory, antioxidant, anticancer, and metabolic modulation effects like insulin sensitivity enhancement and lipid peroxidation reduction [86].

In conclusion, POEE treatment exerted notable antioxidant, anti-inflammatory, and glucose-lowering effects in both the DM and PCOS models. Additionally, in DM rats, POEE showed significant lipid-lowering and hepatoprotective properties. In the PCOS model, it effectively corrected hormonal imbalances, lowered IR, and improved both ultrasonographic and histopathological features of ovarian tissue, highlighting its potential as a promising therapeutic candidate for PCOS management.

This study had several limitations, including a relatively small sample size, a short duration of intervention, and reliance on a single animal model, which may limit the generalizability of the findings to human populations. Additionally, the use of only one extraction method for the plant material may have influenced the concentrations and diversity of the bioactive compounds obtained. As a result, the observed effects may not fully represent the therapeutic potential of the *P. ovata* species. Further research involving varied extraction techniques, longer treatment periods, and clinical trials is needed to confirm and extend these findings.

## 4. Materials and Methods

### 4.1. Chemicals

The materials used in this study were sourced from the following:

**Sigma-Aldrich (Munich, Germany) and Merck (Darmstadt, Germany):** Folin–Ciocâlteu reagent, 2,2-diphenyl-1-picrylhydrazyl (DPPH), sodium nitroprusside, sodium carbonate, aluminium chloride, sodium acetate, methanol, acetate buffer, Griess-Ilosvay nitrite reagent, phosphate-buffered saline, sulphanilic acid, 2,4,6-tri(2-pyridyl)-1,3,5-triazine (TPTZ), ortho-dianisidine dihydrochloride (3,3′-dimethoxybenzidine), thiobarbituric acid, ferric chloride, ethylenediaminetetraacetic acid, xylenol orange, hydrogen peroxide (H_2_O_2_), sodium dodecyl sulphate, 1,1,3,3-tetrahydroxypropane, 5,5′-dithio-bis 2-nitrobenzoic acid (DTNB), butylated hydroxytoluene, Trolox (6-hydroxy-2,5,7,8-tetramethylchroman-2-carboxylic acid), vanadium(III) chloride (VCl_3_), N-(1-Naphthyl) ethylenediamine dihydrochloride (NEDD), HPLC-grade acetonitrile, rutin, and luteolin.

**FineTest Biotech Inc. (Wuhan, Hubei, China):** ELISA kits for NF-κB, IL-1β, 3-NT, 8-OHdG, insulin, testosterone, estrogen, FSH, and LH.

**MyBioSource (San Diego, CA, USA) and ABclonal Technology (Woburn, MA, USA):** Kits for NLRP3 inflammasome biomarkers (IL-18 and Gasdermin)

**Spinreact S.A./S.A.U. (Girona, Spain):** Assay kits for AST, ALT, total cholesterol (TC), triglycerides (TG), and glycemia.

**Sandoz (Basel, Switzerland):** Letrozole.

**Millipore (Burlington, MA, USA):** Ultrapure water (obtained using a Direct-Q UV system).

### 4.2. Extraction and Preparation of P. ovata Ethanol Extract

*P. ovata* seeds were sourced from Springmarkt, Adam Visions (Târgu Mureș, Romania), under batch number AF/4053. The seeds were originally cultivated in the Banaskantha district of Gujarat, India, a region recognized for its optimal agroclimatic conditions for psyllium cultivation, and were subsequently imported into Romania through Poland.

The *P. ovata* seeds (300 g) were ground into a fine powder in a coffee grinder (Argis, RC-21, Electroarges SA, Curtea de Arges, Romania) for 5 min. Further, the plant material was subjected to extraction in the Mycology Laboratory of Babes-Bolyai University, Cluj-Napoca, Romania, using a modified Squibb percolation method. Briefly, the seeds were loaded into three percolators, as follows: 150 g in the first one, 90 g in the second one, and 60 g in the third one. Then, the plant material was soaked with 150 mL of 70% ethanol, and after two days, the three percolated fractions (60 mL, 90 mL, and 120 mL) were collected and mixed. The resulting final extract had a concentration of 1:1 g/mL (*w*/*v*) in 30% ethanol, meaning that 1 g of dry plant material (d.w.) was used per 1 mL of extract. This extract was then diluted with 0.9% saline solution to prepare the dosing solutions, resulting in a concentration of 100 mg d.w./mL (POEE100%). POEE50% and POEE25% were obtained by diluting the POEE100% solution with a 0.9% saline solution in a 1:2 (50%) and 1:4 (25%) ratio, respectively, yielding final concentrations equivalent to 50 mg/mL and 25 mg/mL of dry plant material. All solutions were freshly prepared before administration.

### 4.3. Quantification of Total Phenolic and Flavonoid Content

The TPC of the POEE was assessed using the Folin–Ciocâlteu method, with gallic acid serving as the calibration standard [87]. A reaction mixture was prepared by combining the diluted extract with Folin–Ciocâlteu reagent and distilled water. Subsequently, the volume was adjusted to 25 mL using a 290 g/L sodium carbonate solution, and the mixture was incubated in the dark for 30 min. Absorbance readings were taken at 760 nm with a JASCO V-530 UV-VIS spectrophotometer (Jasco, Tokyo, Japan). The TPC was expressed as mg gallic acid equivalents (GAE) per gram of dry weight, calculated using a gallic acid standard curve (R^2^ = 0.999).

The TFC of POEE was assessed using the aluminum chloride colorimetric method, as previously reported [12]. Briefly, 5 mL of the extract was combined with 5.0 mL of sodium acetate solution (100 g/L) and 3.0 mL of aluminum chloride solution (25 g/L), and the mixture was brought to a final volume of 25 mL using methanol. Absorbance was recorded at 430 nm. TFC was calculated in triplicate and is expressed as milligrams of quercetin equivalents (QE) per gram of dry weight of the plant material, based on a quercetin standard curve (R^2^ = 0.999).

### 4.4. Chromatographic and Mass Spectrometric Profiling

High-performance liquid chromatography (HPLC) was conducted using an Agilent 1200 series system equipped with a diode-array detector (DAD) and coupled to an Agilent 6110 mass spectrometry (MS) detector. Separation was achieved on a Kinetex XB C18 column (4.6 × 150 mm, 5 µm) at 25 °C, using a gradient of water and acetonitrile (both with 0.1% acetic acid) at 0.5 mL/min. Detection was carried out at 280 and 340 nm. MS was operated in positive ESI mode (3000 V, 350 °C, 7 L/min, *m*/*z* 120–1200). Phenolic compounds were quantified using calibration curves prepared in methanol. The method showed a high linearity (R^2^ > 0.99) for rutin and luteolin (with LOD = 0.21 μg/mL and LOQ = 0.64 μg/mL for rutin and LOD = 0.18 μg/mL and LOQ = 0.54 μg/mL for luteolin, respectively).

### 4.5. Evaluation of In Vitro Antioxidant Activity

The antioxidant properties were evaluated through a series of in vitro assays, encompassing DPPH radical scavenging, FRAP, nitric oxide scavenging, and hydrogen peroxide scavenging. All assays were conducted with triplicate samples.

The DPPH assay was carried out by mixing equal volumes of the test solution and a 0.1 mg/mL DPPH solution prepared in methanol, then incubating the mixture in the dark at room temperature for 30 min. Spectrophotometric analysis was performed at 517 nm. The antioxidant capacity is expressed as IC_50_ values (μg/mL) and converted to TX equivalents (TE, μg TE/mL) using a standard calibration curve. Based on the TE values, antioxidant potential was categorized into very good (<50 μg TE/mL), good (50–100 μg TE/mL), weak (100–200 μg TE/mL), or negligible (>200 μg TE/mL) [88,89].

Antioxidant activity was quantified using the ferric-reducing antioxidant power (FRAP) assay, which measures the reduction of Fe^3+^ to Fe^2+^ via TPTZ. A reagent mixture consisting of acetate buffer, TPTZ, and ferric chloride in a 10:1:1 (*v*/*v*/*v*) ratio was incubated for 30 min, after which the absorbance was recorded at 593 nm. The results are expressed as milligrams of Trolox equivalents (TE) per mL [89].

The ability to scavenge nitric oxide (NO) was assessed using the Griess reagent method. The test sample was incubated with sodium nitroprusside (SNP) and phosphate-buffered saline (PBS) for 2.5 h. Subsequently, the resulting mixture was reacted with sulphanilic acid and N-(1-Naphthyl) ethylenediamine dihydrochloride (NEDD). Following a 30 min incubation, absorbance was determined at 546 nm, and the results are expressed as IC_50_ values (mg TE/mL) [90].

The hydrogen peroxide scavenging capacity was determined by combining the test sample in water with a hydrogen peroxide solution. Absorbance was recorded at 230 nm after allowing the reaction to proceed for 10 min. The percentage of H_2_O_2_ quenched was calculated, and the results are presented as IC_50_ values (mg TE/mL of ethanolic plant extract) [91].

### 4.6. Experimental Design

The experimental protocol received ethical approval from the Institutional Animal Ethical Committee (IAEC) of “Iuliu Hațieganu” University of Medicine and Pharmacy, Cluj-Napoca, and was authorized by the National Sanitary Veterinary and Food Safety Authority (approval no. 302/04.04.2022).

This study utilized adult female Wistar albino rats weighing between 200 and 250 g. The animals were maintained in the university’s Animal Facility under standard laboratory conditions, including a 12 h light/dark cycle, a controlled temperature of 22–24 °C, and relative humidity levels ranging from 55% to 60%. They had unrestricted access to standard pelleted feed (Cantacuzino Institute, Bucharest) and water.

For the STZ-induced DM model, the rats were randomly divided into 10 groups (*n* = 8 per group), as follows:

- CONTROL: non-diabetic, untreated.
- STZ: diabetic control group (STZ 55 mg/kg, i.p.) [92].
- STZ + TX: diabetic rats treated with TX (20 mg/kg b.w.) [34].
- STZ + M: diabetic rats treated with metformin (100 mg/100 g b.w.) [91].
- POEE100%, POEE50%, and POEE25%: diabetic rats receiving 100%, 50%, and 25% dilutions of POEE, respectively.

Diabetes was induced by a single intraperitoneal injection of STZ (55 mg/kg) in 0.1 M citrate buffer (pH 4.5) following overnight fasting. The CONTROL group received citrate buffer alone. For 10 consecutive days starting from day one, the CONTROL and STZ groups were administered 1 mL/day of tap water via oral gavage, while the POEE groups received corresponding dilutions of the extract. Beginning on day two, fasting blood glucose levels were monitored daily using an Accu-Check Active glucometer, and insulin aspart (2–3 IU, s.c.) was administered as required.

For the LET-induced PCOS model, rats were divided into five groups (*n* = 8), as follows:

- CONTROL: untreated, healthy control.
- LET: PCOS group receiving LET (1 mg/kg b.w., orally) [93].
- LET + TX: PCOS rats treated with TX (20 mg/kg b.w., orally).
- LET + M: PCOS rats treated with metformin (100 mg/100 g b.w., orally).
- LET + POEE100%: PCOS rats treated with 100% POEE dilution.

LET was administered daily for 21 days in a 0.5% carboxymethyl cellulose (CMC) suspension to induce PCOS. Treatment interventions began on day 10. Ultrasound imaging was performed on day 10 to confirm the presence of polycystic ovarian morphology.

On day 11 (for DM groups) and day 22 (for PCOS groups), under general anesthesia induced with ketamine (70 mg/kg) and xylazine (10 mg/kg), blood samples were obtained via retro-orbital puncture. The collected blood was processed to separate serum, which was then stored at –80 °C until analysis. In the PCOS group, ovaries were harvested post-mortem, weighed, measured, and fixed in 10% phosphate-buffered formalin (pH 7.0) for 24 h in preparation for histological examination. At the end of the experimental period, all animals were euthanized through cervical dislocation in accordance with ethical guidelines.

### 4.7. Pharmacological Studies

#### 4.7.1. Evaluation of the Serum OS Markers

Serum *total oxidative status* (TOS), which reflects overall oxidant levels, was determined using a colorimetric method. This assay relies on the oxidation of ferrous ions (Fe^2+^) to ferric ions (Fe^3+^) by oxidants present in the serum under acidic conditions. The generated ferric ions form a colored complex with xylenol orange, enabling quantification. The procedure was carried out using an automated analyzer, with calibration performed using H_2_O_2_. The results are expressed as μM H_2_O_2_ equivalents/L [94].

Serum *total antioxidant capacity (TAC)*, reflecting overall antioxidant defenses, was determined using a colorimetric method. This assay measures the capacity to neutralize dianisidyl radicals, which are generated by the oxidation of ortho-dianisidine. A standard solution of Fe^2+^-o-dianisidine undergoes a Fenton reaction with hydrogen peroxide, producing hydroxyl radicals. Antioxidants in the sample inhibit the oxidation of o-dianisidine and, thus, reduce color development. The color intensity was measured spectrophotometrically, and the assay was calibrated using TX. TAC values are expressed as mM TX equivalents (TE)/L [95].

The *oxidative stress index (OSI)* involves dividing the TOS value by the TAC and multiplying by 100 to express the result as an arbitrary unit [96].

*SH* concentrations were determined using Ellman’s reagent (5,5′-dithio-bis (2-nitrobenzoic acid)). The reagent reacts with SH groups, and the resulting supernatant absorbance was measured spectrophotometrically at 412 nm. Total SH concentrations are expressed as mM reduced glutathione (GSH)/mL [97].

Serum *malondialdehyde (MDA)* concentration was determined using the thiobarbituric acid reactive substances (TBARS) assay [98]. Briefly, 150 μL of serum was combined with 125 μL of 10% trichloroacetic acid, 125 μL of 5 mM EDTA, 125 μL of 8% sodium dodecyl sulfate, and 10 μL of 0.5% butylated hydroxytoluene. Following incubation and centrifugation, the resulting supernatant was reacted with 500 μL of 0.6% thiobarbituric acid at 95 °C. Absorbance was recorded at 532 nm, and MDA levels were calculated using a standard curve generated from 1,1,3,3-tetraethoxypropane in the range of 0.3–10 nM/mL. Results are expressed as nM of MDA per mL of serum.

*Nitric oxide (NO) levels* were indirectly assessed by quantifying its stable end products, nitrite and nitrate (NOx), using the Griess reaction [99]. Prior to the Griess reaction, nitrates were reduced to nitrites using Vanadium (III) chloride. Serum NOx concentrations were then determined spectrophotometrically and are expressed as μM nitrites/L.

Serum concentrations of *3-Nitrotyrosine (3NT)* and *8-Hydroxydeoxyguanosine (8-OHdG)* were determined using commercially available ELISA kits according to the manufacturers’ instructions. Results are expressed in ng/mL.

*Advanced oxidation protein products (AOPPs)* in serum were determined using a method adapted from Witko-Sarsat et al. [100]. Diluted serum samples were incubated with glacial acetic acid and potassium iodide, and absorbance was recorded at 340 nm to assess the reaction outcome. AOPP concentrations are expressed as µM chloramine-T equivalents/L.

Serum *advanced glycation end product (AGE)* concentrations were determined using a commercial ELISA kit, and the results are reported in U/mL.

#### 4.7.2. Assessment of Serum Inflammatory Biomarkers

Inflammation was assessed by quantifying serum biomarkers of NLRP3 inflammasome activation using ELISA kits, following the manufacturer’s protocols. Specifically, Interleukin-1β (IL-1β), Nuclear Factor-κB p65 (NF-κB p65), and Interleukin-18 (IL-18) levels are expressed in pg/mL, while Gasdermin D levels are expressed in ng/mL.

#### 4.7.3. Evaluation of the Blood Glucose, Lipid Profile, Triglyceride–Glucose Index, Liver Injury, and Anthropometric Parameters

Blood glucose levels were assessed at two time points. Firstly, to confirm hyperglycemia induction by STZ, glucose levels were measured 48 h post-STZ administration using the dorsal tail vein for blood sampling and an ACCU-Check Active glucometer (Roche, Basel, Switzerland) with enzymatic strips. Secondly, venous blood glucose levels were determined at sacrifice (day 11) from retro-orbitally collected samples using commercial assay kits; results are expressed as mg/dL.

Serum levels of total cholesterol (TC) and triglycerides (TG) were determined using commercial assay kits with spectrophotometric methods, in accordance with the manufacturers’ protocols. The results are reported in mg/dL.

The TyG, an established surrogate marker for IR, was calculated at using the formula: TyG = Ln [fasting TG (mg/dL) × fasting glucose (mg/dL)/2] [101].

Liver injury was evaluated by quantifying the serum levels of aspartate aminotransferase (AST) and alanine aminotransferase (ALT) using commercially available assay kits. Enzyme activities are expressed in units per liter (U/L), and the AST/ALT ratio was calculated to provide additional insight into hepatic function.

Body weight (BW) was measured at baseline (day 1) and at study termination (day 11/22), and the change in body weight was calculated and expressed in grams (g).

#### 4.7.4. Hormonal Assays

The serum levels of insulin, FSH (follicle-stimulating hormone), LH (luteinizing hormone), estrogen, and testosterone were assayed via the ELISA technique using commercially supplied kits provided by Finetest (Wuhan, China). ELISA assays were performed according to the manufacturer’s protocols, and absorbance was measured at 450 nm ± 2 nm using a microplate reader.

The Homeostatic Model Assessment of IR (HOMA-IR) was calculated using the formula: [serum glucose (mg/dL) × serum insulin (mIU/mL)]/405 [102].

### 4.8. Histopathological Assessment of Reproductive Organs

In accordance with established ethical protocols, animals underwent humane euthanasia. The ovaries were then isolated, cleared of excess tissue, and washed in saline. Measurements of ovarian weight and diameter were taken. To prepare for histological analysis, the ovaries were fixed in 10% phosphate-buffered formalin (pH 7.0) for a day. Paraffin embedding was conducted using standard histological methods, and thin sections (3 µm) were produced with a microtome. These sections were then stained with Hematoxylin and Eosin (H&E) and visualized using an Olympus BX-51 light microscope (Microscope Central, Willow Grove, PA, USA). Images were captured using an Olympus SC 180 digital camera and analysed using cell Sense software v4.2.1. (Olympus Corporation, Tokyo, Japan) [103].

### 4.9. Ultrasonographic Evaluation

Imaging was conducted utilizing an Esaote MyLab 40 Vet ultrasound system, employing an 18 MHz linear probe. Prior to examination, animals were anesthetized via an intraperitoneal injection of Xylazine (5 mg/kg) and Ketamine (60 mg/kg) [104]. The abdominal area, extending from the costal margin to the caudal region, was shaved bilaterally, and ultrasound gel was liberally applied. Rats were placed in a supine position. On day 10 of the study, two researchers performed the ultrasound scans, working collaboratively. The transducer was systematically manipulated along both the vertical and horizontal planes to visualize and measure ovarian dimensions, as well as to document the appearance and size of ovarian follicles.

### 4.10. Statistical Methods

For data following a normal distribution, results are expressed as the mean ± standard deviation (SD). To determine significant differences between groups, a one-way ANOVA was conducted, followed by Bonferroni–Holm post hoc tests for pairwise comparisons. Pearson correlation analysis was used to assess correlations between biomarkers. Statistical significance was set at *p* < 0.05. Principal Component Analysis (PCA) was utilized for multivariate analysis of the datasets. SPSS v26.0 (SPSS Inc., Chicago, IL, USA) and Statistica 12 v15.0 (TIBCO Software, Palo Alto, CA, USA) software packages were used for all statistical computations.

## 5. Conclusions

This study demonstrated that POEE exerts multiple beneficial effects in experimental models of DM and PCOS. Phytochemical analysis confirmed the presence of key bioactive flavonoids, which contributed to the extract’s observed bioactivities. In vitro antioxidant assays revealed strong radical-scavenging properties of POEE, which were supported by in vivo findings in both disease models. In the STZ-induced diabetic rat model, POEE significantly reduced OS markers, restored antioxidant defenses, and improved metabolic parameters such as glucose, lipid profile, and liver injury enzymes. Moreover, POEE showed anti-inflammatory effects by downregulating NF-κB and NLRP3-inflammasome-related cytokines. In addition to mitigating oxidative damage, POEE also improved metabolic parameters and liver function in STZ-induced T1D rats. In the LET-induced PCOS model, POEE attenuated OS and inflammatory markers while enhancing antioxidant capacity, reducing IR and androgen levels, normalizing the hypothalamic–pituitary–gonadal axis, and improving ovarian morphology, as confirmed by histopathological examination. These effects were dose-dependent and, in many cases, comparable to those of metformin and TX. Overall, the results support POEE as a promising candidate for the complementary management of endocrino-metabolic disorders like DM and PCOS.

## Figures and Tables

**Table 1:** The content of phenolic compounds in *P. ovata* extracts by HPLC (μg/mL).

Peak No.	R_t_ (min)	UV λ_max_ (nm)	[M + H]^+^ (m/z)	Compound	Subclass	Quantity * (μg/mL)
1	12.85	350,255	595,287	Kaempferol-rutinoside	Flavonol	98.695 ± 6.23
2	14.51	355,250	743,303	Quercetin-xylosyl-rutinoside	Flavonol	419.322 ± 25.98
3	15.04	355,250	611,303	Quercetin-rutinoside (Rutin)	Flavonol	259.952 ± 8.34
4	15.39	355,250	465,303	Quercetin-glucoside	Flavonol	225.034 ± 17.11
5	16.12	345,250	611,287	Luteolin-diglucoside	Flavone	36.901 ± 0.78
6	16.97	355,250	479,303	Quercetin-glucuronide	Flavonol	73.309 ± 4.65
7	17.76	350,255	611,287	Kaempferol-diglucoside	Flavonol	86.271 ± 7.43

* Quantity is expressed as μg rutin equivalent/mL for flavonols and μg luteolin equivalent/mL for flavones.

**Table 2:** Free radical scavenging activity of *P. ovata* ethanolic extract.

Sample	DPPH (μgTE/mL)	H_2_O_2_ Scavenging Activity (μgTE/mL)	NO Scavenging Activity (μgQE/mL)	FRAP (mgTE/mL)
*P. ovata* IC50	99.86 ± 4.32	87.06 ± 2.89	121.07 ± 16.22	92.58 ± 5.14
TX IC50	11.2 ± 1.7	24.23 ± 3.12	-	12.07 ± 2.04
Quercetin IC50	-	-	20.58 ± 3.67	-
*p*-value	<0.001	<0.001	<0.001	<0.001

DPPH—α, α-diphenyl-β-picrylhydrazyl; NO—nitric oxide; H_2_O_2_—hydrogen peroxide; FRAP—ferric reducing antioxidant power; QE—quercetin equivalent; TE—Trolox equivalent.

**Table 3:** Effects of *P. ovata* ethanol extract on OS markers in rat STZ-induced DM.

Parameters	Control	STZ	STZ + POEE100%	STZ + POEE50%	STZ + POEE25%	STZ + M	STZ + TX
**TOS** **(μM/L)**	18.11 ± 3.18	40.85 ± 3.18 ^aaa^	15.35 ± 3.18 ^bbb,ccc^	21.43 ± 3.18 ^bbb^	36.98 ± 3.18 ^b^	23.17 ± 3.18 ^bbb^	25.18 ± 11.55 ^bbb^
**TAC (mM/L)**	1.09 ± 0.001	1.084 ± 0.0014 ^a^	1.089 ± 0.001 ^bbb,c^	1.088 ± 0.001 ^bbb,c^	1.09 ± 0.0001 ^bbb^	1.091 ± 0.001 ^bb^	1.09 ± 0.001 ^b^
**OSI**	120.96 ± 2.53	37.67 ± 2.92 ^aaa^	14.08 ± 2.92 ^bbb^	19.47 ± 2.92 ^bbb,cc^	33.25 ± 2.92 ^b^	21.24 ± 2.92 ^bbb^	24.30 ± 12.54 ^b^
**MDA (nM/L)**	2.33 ± 0.04	3.53 ± 0.04 ^aaa^	2.31 ± 0.04 ^bbb^	2.58 ± 0.04 ^bbb^	2.86 ± 0.04 ^bbb^	2.13 ± 0.04 ^bbb^	2.20 ± 0.18 ^bbb^
**AOPP (μM/L)**	140.40 ± 7.77	166.66 ± 7.77 ^aaa^	118.93 ± 7.77 ^bbb,cc,dd^	121.67 ± 7.77 ^bbb,cc,dd^	139.11 ± 7.77 ^b,c,d^	161.59 ± 26.19	166.08 ± 36.79
**8-OHdG (ng/mL)**	38.04 ± 12.36	45.45 ± 12.36 ^a^	23.68 ± 12.36 ^bb,c^	17.80 ± 12.36 ^bb,cc,dd^	30.20 ± 12.36 ^b^	44.63 ± 12.36	41.71 ± 31.66
**AGEs (ng/mL)**	11.18 ± 2.88	14.71 ± 2.88 ^a^	12.42 ± 2.88 ^b^	14.07 ± 2.88 ^cc^	13.42 ± 2.88 ^c^	11.01 ± 2.88 ^bb^	14.76 ± 2.12
**NOx** **(μM/L)**	31.38 ± 5.33	60.58 ± 5.33 ^aaa^	44.48 ± 5.33 ^bb,cc^	42.44 ± 5.33 ^bb,cc^	49.49 ± 5.33	57.83 ± 5.33	58.19 ± 5.45
**3NT (ng/mL)**	36.86 ± 12.28	58.82 ± 12.28 ^a^	21.00 ± 12.28 ^bbb,cc,ddd^	19.27 ± 12.28 ^bbb,cc,ddd^	27.80 ± 12.28 ^bbb,cc,dd^	44.54 ± 12.28 ^b^	64.71 ± 46.28
**SH** **(μM/L)**	322.84 ± 14.47	253.13 ± 14.47 ^a^	387.77 ± 14.47 ^bb,cc,d^	414.63 ± 14.47 ^bb^	254.83 ± 14.47 ^cc,dd^	507.52 ± 150.89 ^bbb^	426.53 ± 109.40 ^bb^

Values are expressed as mean ± SD (standard deviation). vs. CONTROL: ^a^ *p* < 0.05, ^aaa^ *p* < 0.001; vs. STZ: ^b^ *p* < 0.05, ^bb^ *p* < 0.01, ^bbb^ *p* < 0.001; vs. STZ + M: ^c^ *p* < 0.05, ^cc^ *p* < 0.01, ^ccc^ *p* < 0.001; vs. STZ + TX: ^d^ *p* < 0.05, ^dd^ *p* < 0.01, ^ddd^ *p* < 0.001; STZ—Streptozotocin; STZ + TX—Streptozotocin + Trolox; STZ + M—Streptozotocin + Metformin; POEE—*P. ovata* ethanol extract; TOS—total oxidative status; TAC—total antioxidant capacity; OSI—oxidative stress index; MDA: Malonyldialdehide; AOPPs—advanced oxidation protein products; 8-OHdG—8-hydroxydeoxyguanosine; AGEs—advanced glycation end products; NOx—nitrites and nitrates; 3NT—3-nitrotyrosine; SH—total thiols.

**Table 4:** Effects of *P. ovata* ethanol extract on OS markers in rat LET-induced PCOS.

Parameters	Control	LET	LET + POEE100%	LET + M	LET + TX
**TOS (μM/L)**	4.80 ± 0.46	14.40 ± 0.88 ^aaa^	12.29 ± 2.93 ^b,ddd^	13.15 ± 4.41	4.55 ± 0.36 ^bbb^
**TAC (mM/L)**	1.09 ± 0.0001	1.079 ± 0.0001 ^aaa^	1.083 ± 0.0001 ^bbb^	1.09 ± 0.0001 ^bbb^	1.08 ± 0.0001 ^bbb^
**OSI**	4.44 ± 0.34	12.45 ± 1.42 ^aaa^	11.18 ± 1.12 ^b,ddd^	12.43 ± 3.74	4.24 ± 0.28 ^bbb^
**MDA (nM/L)**	2.12 ± 0.31	3.84 ± 0.20 ^aaa^	2.78 ± 0.35 ^bb^	2.96 ± 0.27 ^bbb^	2.46 ± 0.44 ^bbb^
**AOPP (μM/L)**	57.54 ± 4.32	97.64 ± 8.05 ^aaa^	80.64 ± 8.20 ^bb^	77.88 ± 12.16 ^bb^	84.10 ± 12.25 ^b^
**8-OHdG (ng/mL)**	109.05 ± 2.30	148.34 ± 48.09 ^aa^	48.97 ± 14.61 ^bbb,c^	75.53 ± 24.30 ^bb^	37.67 ± 8.75 ^bbb^
**AGEs (ng/mL)**	20.54 ± 10.79	30.69 ± 7.69 ^a^	18.81 ± 6.09	18.39 ± 1.12	15.64 ± 6.20
**NOx (μM/L)**	35.38 ± 5.48	63.89 ± 2.55 ^aaa^	60.00 ± 11.90 ^bb,c^	64.68 ± 7.33	64.73 ± 10.23
**3NT (ng/mL)**	28.24 ± 7.12	59.07 ± 32.99 ^aa^	59.60 ± 31.33	65.70 ± 37.60	51.46 ± 34.43
**SH (μM/L)**	363.83 ± 27.24	304.70 ± 31.11 ^aaa^	319.04 ± 6.70	351.83 ± 41.71 ^b^	355.20 ± 62.51 ^b^

Values are expressed as mean ± SD (standard deviation). vs. CONTROL: ^a^ *p* < 0.05, ^aa^ *p* < 0.01, ^aaa^ *p* < 0.001; vs. LET: ^b^ *p* < 0.05, ^bb^ *p* < 0.01, ^bbb^ *p* < 0.001; vs. LET + M: ^c^ *p* < 0.05; vs. LET + TX: ^ddd^ *p* < 0.001; LET—Letrozole; LET + TX—Letrozole + Trolox; LET + M—Letrozole + Metformin; POEE—*P. ovata* ethanol extract; TOS—total oxidative status; TAC—total antioxidant capacity; OSI—oxidative stress index; MDA: Malonyldialdehide; AOPPs—advanced oxidation protein products; 8-OHdG—8-hydroxydeoxyguanosine; AGEs—advanced glycation end products; NOx—nitrites and nitrates; 3NT—3-nitrotyrosine; SH—total thiols.

**Table 5:** Effects of *P. ovata* ethanol extract on inflammatory markers in rat STZ-induced DM.

Parameters	Control	STZ	STZ + POEE100%	STZ + POEE50%	STZ + POEE25%	STZ + M	STZ + TX
**NFkB-p65 (pg/mL)**	165.37 ± 32.29	314.29 ± 59.94 ^aaa^	196.73 ± 55.44 ^bb,c,d^	231.17 ± 42.75 ^b^	256.67 ± 43.79 ^b^	230.48 ± 18.46 ^b^	223.32 ± 34.26 ^b^
**IL-1B (pg/mL)**	24.38 ± 3.92	132.33 ± 20.18 ^aaa^	19.62 ± 2.66 ^bbb,ddd^	20.85 ± 5.54 ^bbb,d^	26.49 ± 18.77 ^bbb^	19.61 ± 3.93 ^bbb^	27.91 ± 3.15 ^bbb^
**IL-18 (pg/mL)**	17.28 ± 8.12	60.12 ± 10.88 ^aaa^	0.01 ± 0.0002 ^bbb,ccc,ddd^	0.012 ± 0.0001 ^bbb,ccc,ddd^	0.0109 ± 0.0003 ^bbb,ccc,ddd^	11.50 ± 1.28 ^bbb^	12.182 ± 0.55 ^bbb^
**Gasdermin (ng/mL)**	6.002 ± 1.05	9.84 ± 1.27 ^aa^	5.25 ± 0.43 ^bb,cc^	5.77 ± 0.97 ^bb,cc^	6.13 ± 1.04	9.29 ± 1.05	6.51 ± 1.35

Values are expressed as mean ± SD (standard deviation). vs. CONTROL: ^aa^ *p* < 0.01, ^aaa^ *p* < 0.001; vs. STZ: ^b^ *p* < 0.05, ^bb^ *p* < 0.01, ^bbb^ *p* < 0.001; vs. STZ + M: ^c^ *p* < 0.05, ^cc^ *p* < 0.01, ^ccc^ *p* < 0.001; vs. STZ + TX: ^d^ *p* < 0.05, ^ddd^ *p* < 0.001; STZ—Streptozotocin; STZ + TX—Streptozotocin + Trolox; STZ + M—Streptozotocin + Metformin; POEE—*P. ovata* ethanol extract; NFkB-p65—Nuclear Factor Kappa B-p65 subunit; IL-1B—Interleukin-1 Beta; IL-18—Interleukin-18.

**Table 6:** Effects of *P. ovata* ethanol extract on inflammatory markers in rat LET-induced PCOS.

Parameters	Control	LET	LET + POEE100%	LET + M	LET + TX
**NFkB-p65 (pg/mL)**	258.35 ± 48.76	520.89 ± 73.55 ^aa^	281.30 ± 71.59 ^bb,cc^	541.52 ± 93.95	268.39 ± 90.45 ^bb^
**IL-1B (pg/mL)**	24.99 ± 1.60	44.06 ± 10.21 ^aa^	24.43 ± 3.66 ^bb^	22.17 ± 3.35 ^bb^	27.15 ± 0.89 ^bb^
**IL-18 (pg/mL)**	19.47 ± 2.48	33.20 ± 7.30 ^aa^	17.76 ± 3.67 ^bb^	16.03 ± 1.08 ^bbb^	20.92 ± 6.16 ^b^
**IL-10 (ng/mL)**	15.79 ± 2.19	29.66 ± 5.81 ^aaa^	16.01 ± 2.25 ^bbb,dd^	14.44 ± 2.77 ^bbb^	23.00 ± 1.46 ^b^

Values are expressed as mean ± SD (standard deviation). vs. CONTROL: ^aa^ *p* < 0.01, ^aaa^ *p* < 0.001; vs. LET: ^b^ *p* < 0.05, ^bb^ *p* < 0.01, ^bbb^ *p* < 0.001; vs. LET + M: ^cc^ *p* < 0.01; vs. LET + TX: ^dd^ *p* < 0.01; LET—Letrozole; LET + TX—Letrozole + Trolox; LET + M—Letrozole + Metformin; POEE—*P. ovata* ethanol extract; NFkB-p65—Nuclear Factor Kappa B-p65 subunit; IL-1B—Interleukin-1 Beta; IL-18—Interleukin-18; IL-10—Interleukin-10.

**Table 7:** Antidiabetic, antihyperlipidemic, and hepatoprotective activities of *P. ovata* ethanol extract in rat STZ-induced DM.

Parameters	Control	STZ	STZ + POEE100%	STZ + POEE50%	STZ + POEE25%	STZ + M	STZ + TX
**GLU (mg/dL)**	102.91 ± 8.39	426.76 ± 26.71 ^aaa^	360.51 ± 38.69 ^bb^	411.98 ± 36.79	422.25 ± 56.36	370.72 ± 49.08 ^bb^	412.5 ± 139.36
**TG (mg/dL)**	66.10 ± 23.86	129.26 ± 6.15 ^aaa^	92.96 ± 19.59 ^b,ccc,dd^	102.99 ± 12.25 ^b,cc,d^	113.00 ± 12.41 ^c,d^	133.09 ± 5.33	116.62 ± 7.53 ^b^
**TyG index**	4.38 ± 0.12	5.46 ± 0.11 ^aa^	5.20 ± 0.11 ^b^	5.32 ± 0.65 ^bb^	5.38 ± 0.13	5.40 ± 0.06	5.38 ± 0.11
**TC (mg/dL)**	72.97 ± 12.33	123.51 ± 8.21 ^aaa^	72.95 ± 12.09 ^bbb,ccc^	98.03 ± 12.12 ^bbb^	105.83 ± 6.99 ^bbb,c^	98.06 ± 12.27 ^bbb,dd^	112.36 ± 12.75
**AST (U/L)**	67.51 ± 15.04	156.8 ± 16.04 ^aaa^	119.06 ± 34.73 ^bb^	114.78 ± 20.13 ^bb^	125.61 ± 25.35	125.42 ± 24.38	131.07 ± 28.54
**ALT (U/L)**	62.87 ± 14.38	159.92 ± 18.24 ^aaa^	104.18 ± 32.003 ^bb^	117.48 ± 22.41 ^bb^	130.55 ± 24.53	126.76 ± 16.03 ^b^	137.79 ± 42.05
**AST/ALT**	1.08 ± 0.10	0.98 ± 0.03 ^aa^	1.04 ± 0.09	0.98 ± 0.18	0.96 ± 0.09	0.98 ± 0.18	0.94 ± 0.102
**BW (g) change**	15.14 ± 3.30	104.16 ± 26.62 ^aaa^	33.54 ± 13.46 ^bb^	64.63 ± 20.03 ^b,cc^	67.39 ± 22.46 ^b,cc^	27.39 ± 15.01 ^bb^	56.25 ± 18.55 ^bb^

Values are expressed as mean ± SD (standard deviation). vs. CONTROL: ^aa^ *p* < 0.01, ^aaa^ *p* < 0.001; vs. STZ: ^b^ *p* < 0.05, ^bb^ *p* < 0.01, ^bbb^ *p* < 0.001; vs. STZ + M: ^c^ *p* < 0.05, ^cc^ *p* < 0.01, ^ccc^ *p* < 0.001; vs. STZ + TX: ^d^ *p* < 0.05, ^dd^ *p* < 0.01; STZ—Streptozotocin; STZ + TX—Streptozotocin + Trolox; STZ + M—Streptozotocin + Metformin; POEE –*P. ovata* ethanol extract; AST—aspartate amino-transferase; ALT—alanine amino-transferase; AST/ALT—aspartate to alanine amino-transferase ratio; TC—total cholesterol; TG—triglyceride; GLU—glucose; TyG index—Triglyceride to Glucose index; BW—body weight.

**Table 8:** Effects of *P. ovata* on hormonal markers in rat LET-induced PCOS.

Parameters	Control	LET	LET + POEE100%	LET + M	LET + TX
**Insulin (pg/mL)**	30.15 ± 6.63	99.53 ± 12.59 ^aaa^	24.08 ± 4.30 ^bbb^	25.81 ± 4.85 ^bbb^	27.89 ± 8.44 ^bbb^
**HOMA-IR**	7.91 ± 3.34	32.59 ± 8.86 ^aaa^	5.74 ± 4.38 ^bbb^	7.11 ± 4.43 ^bbb^	7.14 ± 2.41 ^bbb^
**GLU (mg/dL)**	100.97 ± 19.57	129.45 ± 12.59 ^a^	77.69 ± 14.20 ^bbb,c,d^	104.49 ± 5.94 ^b^	108.92 ± 18.02 ^b^
**FSH (mIU/ml)**	39.19 ± 4.48	99.86 ± 33.16 ^aa^	80.39 ± 18.30 ^c^	99.33 ± 3.70	101.13 ± 34.50
**LH (mIU/ml)**	9.97 ± 4.66	26.05 ± 10.10 ^aa^	14.60 ± 5.87 ^b,c^	20.69 ± 14.05	13.12 ± 2.97 ^b^
**Estrogen (pg/mL)**	596.34 ± 67.74	329.88 ± 55.80 ^aaa^	728.07 ± 166.13 ^bbb^	774.90 ± 55.74 ^bbb^	621.89 ± 166.06 ^bbb^
**Testosterone (ng/mL)**	0.76 ± 0.24	1.17 ± 0.83 ^a^	1.31 ± 0.17 ^b^	1.55 ± 1.05	2.44 ± 0.75

Values are expressed as mean ± SD (standard deviation). vs. CONTROL: ^a^ *p* < 0.05, ^aa^ *p* < 0.01, ^aaa^ *p* < 0.001; vs. LET: ^b^ *p* < 0.05, ^bbb^ *p* < 0.001; vs. LET + M: ^c^ *p* < 0.05; vs. LET + TX: ^d^ *p* < 0.05; LET—Letrozole; LET + TX—Letrozole + Trolox; LET + M—Letrozole + Metformin; POEE—*P. ovata* ethanol extract; GLU—blood glucose; FSH—follicle-stimulating hormone, LH—luteinizing hormone.

## Data Availability

The data presented in this study are available on request from the corresponding author.

## Abbreviations

The following abbreviations are used in this manuscript:

DM	Diabetes Mellitus
PCOS	Polycystic ovary syndrome
POEE	*Plantago ovata* ethanol extract
STZ	Streptozotocin
LET	Letrozole
OS	Oxidative stress
ROS	Reactive oxygen species
TX	Trolox
TPC	Total phenolic content
TFC	Total flavonoid content
TOS	Total oxidative status
TAC	Total antioxidant capacity
OSI	Oxidative stress index
MDA	Malondialdehyde
AOPPs	Advanced oxidation protein products
8-OHdG	8-Hydroxydeoxyguanosine
NO	Nitric oxide
3-NT	3-Nitrotyrosine
AGEs	Advanced glycation end products
SH	Total thiols
NF-kB-p65	Nuclear Factor Kappa B-p65
IL-1B	Interleukin-1 Beta
IL-18	Interleukin-18.
IL-10	Interleukin-10
GLU	Blood glucose
LH	Luteinizing hormone
FSH	Follicle-stimulating hormone
TyG	Triglyceride–Glucose index
IR	Insulin resistance
HOMA-IR	Homeostatic Model Assessment for Insulin Resistance
